# Dr. Bentley S. Bourne

**Published:** 1916-02

**Authors:** 


**Dr. Bentley S. Bourne,** Niagara 1890, Health Physician of the village of Hamburg, N. Y., died at the residence of his brother, Charles L. Bourne, in Salisbury, Md., Dec. 25, 1915. He was born in Boston, N. Y., Sept. 16, 1866. He studied at the Central High School of Buffalo. He had practiced medicine in Hamburg for 25 years. He was a member of Fraternal Lodge, No. 625, F. & A. M., and of Hamburg Lodge of the I. O. O. F. The funeral was held in Hamburg, Dec. 28.

